# Genetic diversity in fishes is influenced by habitat type and life‐history variation

**DOI:** 10.1002/ece3.4661

**Published:** 2018-11-11

**Authors:** Alexander S. Martinez, Janna R. Willoughby, Mark R. Christie

**Affiliations:** ^1^ Department of Biological Sciences Purdue University West Lafayette Indiana; ^2^ Department of Forestry and Natural Resources Purdue University West Lafayette Indiana

**Keywords:** climate change, fisheries, genetic variation, microsatellites

## Abstract

Populations of fishes are increasingly threatened by over‐exploitation, pollution, habitat destruction, and climate change. In order to better understand the factors that can explain the amount of genetic diversity in wild populations of fishes, we collected estimates of genetic diversity (mean heterozygosity and mean rarefied number of alleles per locus) along with habitat associations, conservation status, and life‐history information for 463 fish species. We ran a series of phylogenetic generalized least squares models to determine which factors influence genetic diversity in fishes after accounting for shared evolutionary history among related taxa. We found that marine fishes had significantly higher genetic diversity than freshwater fishes with marine fishes averaging 11.3 more alleles per locus than their freshwater counterparts. However, contrary to our expectations, genetic diversity was not found to be lower in threatened versus not‐threatened fishes. Finally, we found that both age at maturity and fecundity were negatively related to genetic variation in both marine and freshwater fishes. Our results demonstrate that both life‐history characteristics and habitat play a role in shaping patterns of genetic diversity in fishes and should be considered when prioritizing species for conservation.

## 1 INTRODUCTION

Fishes are the most speciose group of vertebrates (over 33,000 species described), inhabit nearly all major aquatic habitat types, and perform a diverse set of biological functions in ecosystems (Helfman, Collette, Facey, & Bowen, 2009). However, fishes are increasingly faced with altered environmental conditions and disturbance resulting from human activity. In particular, overharvesting, habitat destruction, pollution, and the introduction of non‐native species have led to a global decline in marine and freshwater fish biodiversity (Leidy & Moyle, 1998; Pauly & Zeller, 2016). Furthermore, fishes increasingly need to contend with the effects of global climate change, which is driving ocean acidification and increases in aquatic temperatures and is expected to impose regional changes to salinity, dissolved oxygen availability, and circulation patterns in aquatic environments (Crozier & Hutchings, 2014; Levitus et al., 2012G; O’Reilly et al., 2015; Pachauri et al., 2014). Thus, disentangling species that are likely to adapt to future environmental changes from those that will require intervention remains a fundamental challenge for successful conservation and management of fishes.

One metric that may help predict which species are most likely to adapt to future conditions is genetic diversity. Broadly speaking, genetic diversity is any measure that quantifies within‐population variability in alternative forms of genes or noncoding loci (Hughes, Inouye, Johnson, Underwood, & Vellend, 2008). The ability of a population to evolve and adapt may be related to both heterozygosity (i.e., the proportion of diploid individuals that have two different alleles at a single locus) and the total number of alleles present within a population (Allendorf, 1986; Frankham, Bradshaw, & Brook, 2014). Additionally, reduced genetic diversity may result in decreased population viability and increased extinction likelihood, particularly for populations faced with stressful environmental conditions (Markert et al., 2010; Vandewoestijne, Schtickzelle, & Baguette, 2008; reviewed in Reed & Frankham, 2003). Understanding how patterns of genetic diversity vary across fishes could help inform predictions regarding which species are likely to adapt in response to future disturbance while simultaneously identifying species that might be particularly susceptible to extinction (Reed & Frankham, 2003; Stockwell, Hendry, & Kinnison, 2003).

A landmark study by DeWoody and Avise (2000) analyzed 32 fish species and provided one of the first quantitative comparisons of genetic diversity in fishes occupying different habitats. The study suggested that genetic diversity was higher in marine fishes relative to freshwater fishes. We have since identified hundreds of additional studies that directly estimate genetic diversity in 463 distinct fish species, providing a much broader taxonomic survey and increased power and precision for statistical analyses. Furthermore, improvements to phylogenies and statistical methods that consider evolutionary relatedness among species now allow us to account for the confounding effect of shared evolutionary histories and ask new questions about potential drivers of patterns of genetic diversity in fishes (Pennell, 2014; Revell, 2010).

In this study, we ask how genetic diversity relates to species habitat needs, conservation status, and life‐history characteristics. We have three predictions. First, following DeWoody and Avise (2000), we predict that marine fishes should generally have higher genetic diversity than freshwater fishes even after accounting for species relatedness. Second, increased conservation need, as determined by the International Union for Conservation of Nature (IUCN), is determined in part by a reduction in census population size (IUCN, 2018), which is often associated with a reduction in genetic diversity. Therefore, we predict that threatened species (i.e., species of high conservation concern) are likely to have less genetic diversity compared to not‐threatened species (sensu Willoughby et al., 2015). Finally, certain life‐history characteristics can influence genetic diversity across a wide variety of taxa (Frankham et al., 2014; Romiguier et al., 2014; Waples, Luikart, Faulkner, & Tallmon, 2013). Romiguier et al. (2014) showed that species exhibiting life‐history characteristics typically associated with “r‐strategists,” including both early age at maturity and high fecundity, tend to have more genetic diversity than species that mature later and have fewer offspring (hereafter referred to as “K‐strategists”). As a result, we predict that genetic diversity should decrease as age at maturity increases in fishes. Additionally, given that r‐strategists tend to exhibit high fecundity, we predict that genetic diversity will increase with fecundity in fishes. To identify patterns and drivers of genetic diversity in fishes, we test each of these predictions by determining the relationship between genetic diversity and (i) habitat, (ii) conservation status, and (iii) life‐history characteristics by performing a quantitative review of 463 globally distributed fish species.

## 2 METHODS

### 2.1 Data collection

We collected estimates of genetic diversity, taxonomic relationships, habitat information, conservation status, and life‐history characteristics for a diverse array of fishes. We first performed Web of Science and Google Scholar title, keyword, and abstract searches including the search terms “microsatellite” and “fish” and excluding the terms “cancer” and “fluorescence in situ hybridization” (i.e., FISH) to filter out medical journals unrelated to genetic diversity in fishes. We focused on microsatellite loci because 1. far fewer studies to date have used alternative nuclear markers to measure genetic diversity in fishes (e.g., there are fivefold fewer search results for “fish” and “single nucleotide polymorphisms” when employing the same exclusions used to search for microsatellite studies) and 2. we wanted to make comparisons across similar marker types to avoid confounding factors (e.g., total number of alleles can only vary from 1 to 4 in SNPs). To expedite data entry, we cross‐referenced our search results against a similar dataset amassed for all vertebrates (Willoughby et al., 2015). For species with multiple publications estimating genetic diversity, we retained only the study with the highest sample size (i.e., number of individuals). Additionally, we extracted data from the single largest population when multiple populations were sampled in a single study. Our final dataset generally included studies which had estimates of both mean observed heterozygosity (i.e., heterozygosity averaged across loci; hereafter “heterozygosity”) and mean number of alleles per locus, although a small number of studies (*n* = 4) lacked estimates of heterozygosity. In sum total, we ended up with 463 studies, each corresponding to a distinct species (Supporting information Appendix S1).

In our dataset, sample sizes were highly variable and ranged from 10 to 974 individuals (μ = 77.4 ± 27.32 SE). Because the number of microsatellite alleles identified per locus is positively related to the number of individuals sampled (Kalinowski, 2004; Mousadik & Petit, 1996), we performed allele rarefaction using a quasi‐maximum‐likelihood approach. This modified approach was required (i.e., we could not use established ML methods such as (Kalinowski, 2004)) because many studies that recorded the total number of alleles did not report the per‐locus allele frequencies. For each study, we first tested the total number of alleles reported in the paper. We created an allele frequency distribution using a set of alleles whose frequencies were determined by the equation:

$$z=\frac{i}{\left(\text{Na}+1\right)-i}\cdot \sum_{i=1}^{\text{Na}}\frac{i}{\left(\text{Na}+1\right)-i}$$

where Na equals the total number of alleles and *i* equals allele *i* in the set 1:Na. This distribution is conservative as it results in several fairly common alleles (Bernatchez & Duchesne, 2000; Christie, 2010). From this allele frequency distribution, we created 1,000,000 genotypes in Hardy–Weinberg Equilibrium. We next took a sample, without replacement, from this large population with a sample size equal to the total number of individuals genotyped in the study of interest and calculated the total number of alleles found within the sample. We repeated this sampling effort 100 times and each time recorded the total number of alleles found in the sample. We then calculated the difference between the mean number of alleles sampled and the number of alleles used to create the 1,000,000 genotypes (equal to total number of alleles reported in the study for this first iteration).

We next iteratively repeated the entire process for Na+1, Na+2, Na+3…. and for Na−1, Na−2, Na−3…. where Na equals the total number of alleles reported in the study. We took the estimate of Na that minimized the absolute value of the difference between mean number of alleles from the 100 simulated samples and the Na being tested (hereafter: “rarefied” number of alleles [Supporting information Figure S1]). The procedure occurred iteratively and when a new minimum difference was found we always tested ±10 additional alleles to ensure that the true estimate was found (note that Na = 2 was set as the minimum, but there was no maximum). This procedure resulted in an average change in the estimated number of alleles by a value of 6.04 alleles, although this change was most substantial for studies that had small sample sizes and high estimates of total numbers of alleles (Supporting information Figure S2). We report results with both the rarified and unadjusted total number of alleles.

We next supplemented our genetic data with taxonomic, habitat, and life‐history data for each species. First, phylogenetic relationships were approximated using taxonomic classifications (i.e., Class, Order, Family, Genus, specific epithet) from Nelson, Grande, and Wilson (2016). Although the use of branch lengths would have improved the resolution of relatedness among taxa, this information was not available for the species we included in our dataset so we relied on taxonomy as a proxy. Next, we obtained species’ habitat information using FishBase (Froese & Pauly, 2017). For each species included in our dataset, we recorded the dominant habitat type (either marine or freshwater) that each species occupied. Additionally, we searched primary literature to identify species habitat requirements when FishBase did not contain relevant data. In some cases, species could not be classified into a single habitat (*e.g.,* anadromous salmonids, brackish‐water fishes) and these species were categorized into a third category (“mixed”). Next, we determined species’ conservation need using IUCN Red List designations for each species (IUCN, 2018). Based on IUCN category definitions, we considered species listed as *least concern* or *near threatened* as “not‐threatened” and species listed as *vulnerable*,* endangered*, or *critically endangered* as “threatened.” Species listed as *extinct* or *extinct in the wild* were excluded from our dataset, while species classified as *not evaluated* or *data deficient* were excluded only from models investigating the relationship between genetic diversity and conservation status. Finally, we again used FishBase to collect data for two life‐history traits for species in our dataset: minimum age at maturity and the maximum absolute fecundity (i.e.*,* the total number of eggs produced by a female).

### 2.2 Statistical analyses

We used three sets of statistical models to understand how genetic diversity varied in fishes that (a) occupied different habitats, (b) were characterized by differing levels of conservation need, and (c) had different life‐history characteristics. For each model set, we considered the effects of our predictor variables (i.e., dominant habitat, conservation status, age at maturity, and fecundity) on both heterozygosity and the rarefied mean number of alleles for each species. We analyzed our genetic, taxonomic, habitat, and life‐history data simultaneously using Phylogenetic Generalized Least Squares (PGLS) regressions using the NLME package in R (Pinheiro, Bates, DebRoy, Sarkar, & R Core Team, 2017). These regressions account for nonindependence of observations in our dataset (i.e., species’ traits) resulting from shared evolutionary history by calculating covariances among traits assuming a Brownian evolution model (Pennell, 2014). For each PGLS model (described in detail below), we used bootstraps (10,000 iterations) to generate confidence intervals around the PGLS coefficient estimates. Within each PGLS model, we identified significant coefficient estimates as those that had bootstrapped 95% CIs (around the mean coefficient estimates) that did not overlap zero. Furthermore, we identified significant differences between groups by comparing the 95% CIs (around the mean coefficient estimates) between groups for a given model; nonoverlapping CIs indicated that coefficient estimates for two groups were significantly different.

For our first set of PGLS models, we assessed the relationship between habitat and genetic diversity by comparing freshwater and marine fishes using the model *Y_i_* = *ß*_1_ * habitat*_i_* + *ε_i_*, where *Y_i_* represents an estimate of genetic diversity, habitat*_i_* corresponds to fishes inhabiting either marine or freshwater habitats, and *ε_i_* equals the error (described below). Next, we analyzed the effect of conservation status (i.e.*,* threatened vs. not‐threatened species) on genetic diversity for fishes within each major habitat type (i.e.*,* freshwater or marine). PGLS models assessing the relationship between conservation status and genetic diversity were run separately for marine and freshwater fishes using the model *Y*_i_ = *ß*_1_ * conservation status*_i_* + *ε*_i_. Finally, we considered the effects of two life‐history traits—age at maturity and fecundity—on genetic diversity. For these life‐history models, we analyzed each life‐history parameter separately, and ran separate models for the two habitat groups (i.e., freshwater and marine). Analyzing all factors together (i.e., all levels of the factor habitat along with a life‐history trait) resulted in overdispersion. The general model used for analyzing the relationship between life‐history variables and genetic diversity was *Y_i_* = *ß*_1_ * life history*_i_* + *ε_i_*, where life history*_i_* represents each of our life‐history variables of interest for the *i* ^th^ species. For each model, the error term *ε_i_* can be thought of as *ε* ~ *N*(0, *V*σ^2^), where *V* is the covariance matrix containing estimates of shared evolutionary history between pairs of species and σ^2^ is the standard deviation (Pennell, 2014). All of our statistical models were run in R (version 3.4.2 –R Core Team, 2017), and all R code is available via GitHub ( https://github.com/ChristieLab/Fish_GD_Echology_Evolution).

## 3 RESULTS

**Figure 1:**
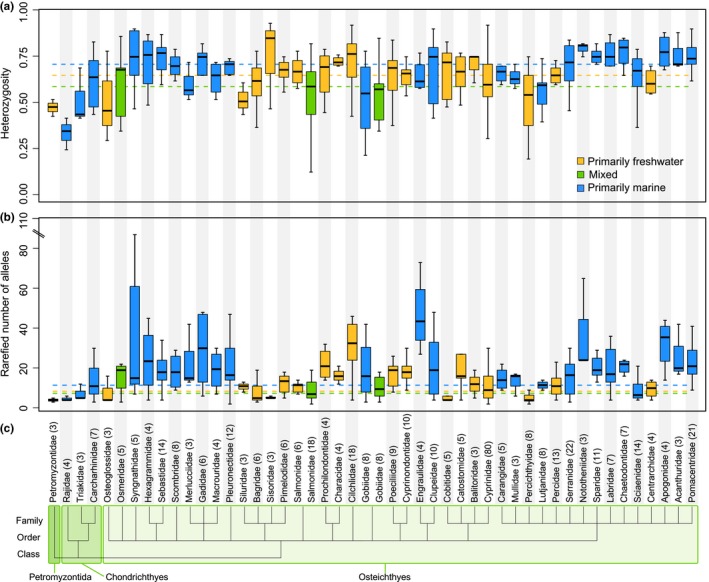
Mean genetic diversity estimates across families of fishes from different habitats. Mean heterozygosity (a) and rarefied mean number of alleles per locus (b) are represented across families of fishes for all families with at least three species in our dataset. Taxonomic relatedness is indicated by the tree (c), where the number of species in each family is noted at each branch tip in parentheses after the family name. Median genetic diversity across species within each habitat type are represented by dashed lines

In total, our final dataset included 463 species across three classes (*N* = 426 in Osteichthyes, *N* = 32 in Chondrichthyes, and *N* = 5 in Petromyzontida), 19 orders, and 46 families of fishes. Marine and freshwater species were represented equally with 215 and 204 species, respectively, whereas mixed species represented a much smaller proportion of our dataset (44 species; Table 1). For subsequent models that rely on PGLS regressions, our dataset was trimmed to include only species within the class Osteichthyes due to small sample sizes in classes Chondrichthyes and Petromyzontida, although life‐history patterns for Chondrichthyes are shown in Supporting information Figure S3. Estimates of mean heterozygosity and rarefied mean number of alleles were estimated from a per‐study average of 14 microsatellite loci (range: 4–300). Among‐family estimates of heterozygosity and rarefied mean number of alleles varied by a factor of 2.3 and 11.69, respectively (Figure 1). Estimates of unadjusted (i.e., not rarefied) mean number of alleles (Supporting information Figure S4) showed the same general patterns (cf Figure 1 and Supporting information Figure S4) although there was variation in point estimates among families. Families with the highest genetic diversity include Nototheniidae (mean heterozygosity = 0.79 ± 0.02 SE) and Engraulidae (rarefied mean number of alleles per locus = 46.75 ± 9.63), whereas the families with the lowest genetic diversity include Rajidae (mean heterozygosity = 0.34 ± 0.03) and Petromyzontidae (rarefied mean number of alleles per locus = 4 ± 0.58).

**Table 1:** Taxonomic breakdown including the number of orders, families, and species of fishes from each of three habitats in our dataset. Not‐threatened species included those listed as either *least concern* or *near‐threatened* by the International Union of the Conservation of Nature while Threatened species included species listed as *vulnerable*,* endangered*, or *critically endangered*

Habitat	Orders	Families	Species	Not‐threatened	Threatened
Freshwater	15	28	204	88	43
Marine	16	41	215	98	33
Mixed	9	11	44	24	6

**Figure 2:**
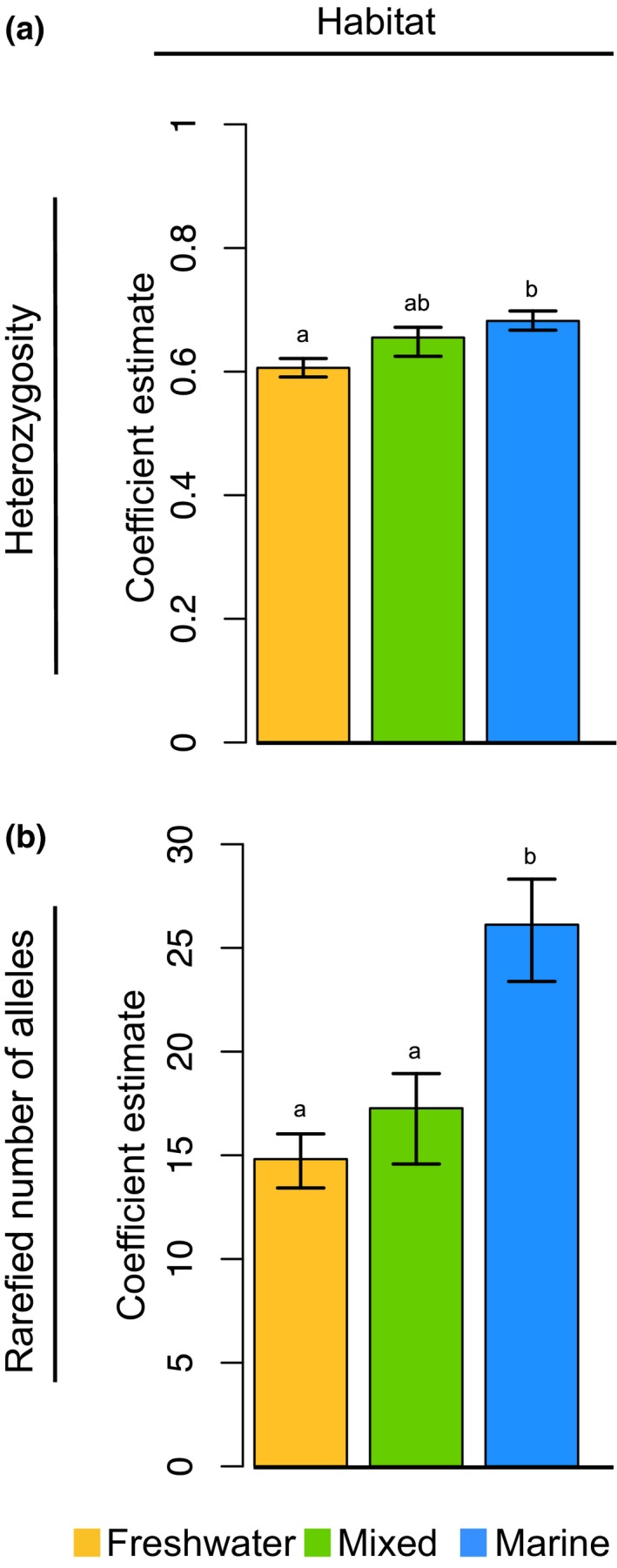
Bootstrapped phylogenetic least squares regression coefficients of mean heterozygosity (a) and rarefied mean number of alleles (b) across habitats in fishes. Error bars represent 95% confidence intervals generated via bootstrapping. Significant relationships (nonoverlapping CIs between habitats) are represented by distinct letters (i.e., a and b). These data illustrate that marine species have higher genetic diversity than freshwater fishes

After accounting for taxonomic relatedness, we found that marine fishes had higher genetic diversity than freshwater fishes. Coefficient estimates from the PGLS habitat models represent the taxonomically corrected mean estimates of genetic diversity and associated variance for fishes within each habitat group. Marine fishes had significantly higher heterozygosity (marine coef: 0.68, 95% CI [0.67, 0.70]; freshwater coef: 0.61, 95% CI [0.59, 0.62]) and more alleles per locus (marine coef: 26.12, 95% CI [23.38, 28.32]; freshwater coef: 14.81, 95% CI [13.42, 16.03]) relative to freshwater species (Figure 2). Mixed species generally fell between the marine and freshwater species estimates (Figure 2).

**Figure 3:**
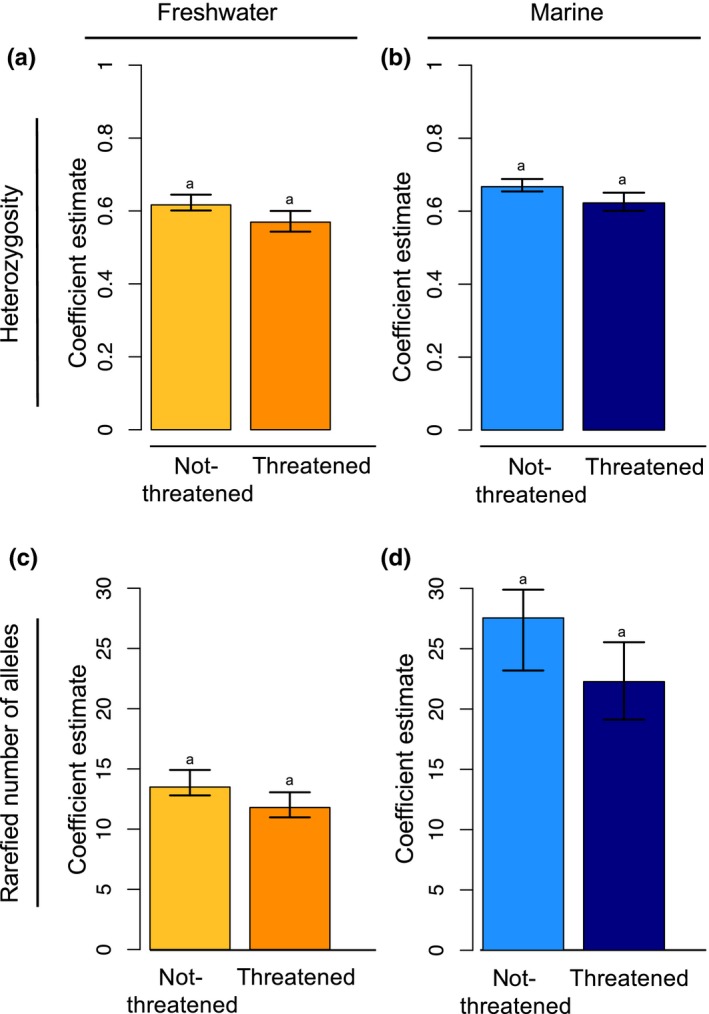
Bootstrapped phylogenetic least squares regression coefficients of mean heterozygosity and rarefied mean number of alleles per locus in freshwater (a and c) and marine (b and d) fishes, estimated across species conservation need. Error bars represent 95% confidence intervals generated via bootstrapping. Significant relationships (nonoverlapping CIs between habitats) are represented by distinct letters (i.e., a and b). These data illustrate that threatened and not‐threatened fishes have similar levels of genetic diversity regardless of habitat

Of the species included in our dataset, 283 had been assessed by the IUCN; we identified 194 not‐threatened and 69 threatened species (with 20 species classified as either *data deficient* or *extinct*; Table 1). Again, the coefficient estimates from the PGLS conservation status models represent the taxonomically corrected mean estimates of genetic diversity and associated variance for each habitat‐conservation status group. In our second PGLS model, we found no difference in genetic diversity between threatened and not‐threatened fishes from either freshwater (heterozygosity—not‐threatened coef: 0.62, 95% CI [0.60,0.65]; threatened coef: 0.57, 95% CI [0.54, 0.60]; rarefied mean number of alleles—not‐threatened coef: 13.50, 95% CI [12.80,14.92]; threatened coef: 11.80, 95% CI [10.98, 13.06]) or marine habitats (heterozygosity—not‐threatened coef: 0.67, 95% CI [0.65, 0.69]; threatened coef: 0.62, 95% CI [0.60, 0.65]; rarefied mean number of alleles—not‐threatened coef: 27.56, 95% CI [23.20, 29.90]; threatened coef: 22.27, 95% CI [19.12, 25.54]) (Figure 3). Despite the lack of significance, in all cases the mean estimates of genetic diversity were lower for threatened fishes than for non‐threatened fishes (both freshwater and marine; Figure 3).

Finally, minimum age at maturity and maximum fecundity estimates were available for 263 and 198 species, respectively. Here, the coefficient estimates from the PGLS conservation status models represent the slope of the regression line where each life‐history trait was regressed against each measure of genetic diversity (e.g., a negative coefficient estimate means that mean heterozygosity decreases as minimum age at maturity increases). We found that as age at maturity and fecundity increased in marine fishes, genetic diversity decreased. For marine species, age at maturity was negatively related to the rarefied mean number of alleles (coef: −0.96, 95% CI [−1.70, −0.43]), although this trend was not significant for heterozygosity (coef: −0.002, 95% CI [−0.006, 0.001]). Fecundity was negatively related to both heterozygosity (coef: −0.01, 95% CI [−0.02, −0.01]) and mean rarefied number of alleles (coef: −0.97, 95% CI [−1.70, −0.43]). We found similar patterns for freshwater fishes (rarefied mean number of alleles—age at maturity coef: −0.82, 95% CI [−1.22, −0.35]; fecundity coef: −0.51, 95% CI [−0.73, −0.38]) with the exception of heterozygosity (age at maturity coef: −0.02, 95% CI [−0.03, 0.001]; fecundity coef: (0.003, 95% CI [−0.002, 0.006]).

## 4 DISCUSSION

After accounting for taxonomic relationships, marine fishes had substantially greater genetic diversity than freshwater fishes. Remarkably, marine fishes averaged 11.3 more alleles per locus than freshwater fishes after standardizing for differences in sample size. Higher genetic diversity in marine fishes is likely attributable to differences in the frequency, magnitude, and interactions between genetic drift and gene flow. Lower genetic drift in marine species could be part of the explanation, especially in light of the fact that population sizes are typically orders of magnitude larger in marine fishes relative to freshwater fishes (Gregory & Witt, 2008; Ward, Woodwark, & Skibinski, 1994). Because effective population size (*N_e_*) is often positively correlated with census population size, larger population sizes in marine fishes should generally translate to larger *N_e_* and therefore higher genetic diversity relative to freshwater species (Hauser & Carvalho, 2008). Large populations characteristic of marine species may also reflect larger range sizes and also suggest that marine fishes may inhabit more productive environments that allow for higher carrying capacities. Lastly, larger population sizes also suggest that marine environments may be more stable and thus may be less sensitive to or experience smaller amounts of genetic drift (April, Hanner, Dion‐Côté, & Bernatchez, 2012; DeWoody & Avise, 2000).

However, given that (a) the ratio between *N_e_* and census estimates $\left(i\text{.e.},\frac{N_{e}}{N}\right)$ can vary widely between species and (b) *N_e_* can be orders of magnitude smaller than census population sizes for some marine fishes (Hauser, Adcock, Smith, Bernal Ramírez, & Carvalho, 2002; Waples et al., 2013), large population sizes alone may not be sufficient to explain these patterns. One alternative possibility is that the high gene flow typically found in marine species buffers against the effects of genetic drift. In marine species, larval dispersal connects local populations, which together form large marine metapopulations (Kritzer & Sale, 2004), and high population connectivity increases genetic diversity. Furthermore, because of the high population connectivity found in marine systems, discrete populations may not always be sampled; sampling cohorts of recruits originating from multiple populations could therefore increase genetic diversity estimates of some marine species in our study. Finally, discrete reproductive events occurring throughout a single breeding season or across several breeding seasons within a population can result in multiple cohorts at a single site originating from different parents; while genetic diversity within each cohort may be low, studies sampling among such cohorts would detect higher levels of genetic diversity. Freshwater environments, by contrast, often have lower levels of gene flow and consequently the effects of genetic drift can be exacerbated (Thomaz, Christie, & Knowles, 2016). Given that microsatellite markers are typically neutral (although see: Chapman, Nakagawa, Coltman, Slate, & Sheldon, 2009; Coltman & Slate, 2007; Forstmeier, Schielzeth, Mueller, Ellegren, & Kempenaers, 2012; Reed & Frankham, 2003), we suggest that this result cannot be explained by differences in selection between freshwater and marine environments. Differences in rates of mutation between marine and freshwater environments are an additional, although unlikely, mechanistic explanation given that our study surveyed a diverse array of taxonomic groups. Regardless of the mechanism, marine fishes have substantially greater genetic diversity than their freshwater counterparts—an observation that should be considered within the context of their continued conservation and management.

We predicted that species listed as threatened by the IUCN should have lower genetic diversity than non‐threatened species. However, we found no significant difference in genetic diversity between conservation categories in either marine or freshwater habitats, as coefficient estimates of both mean heterozygosity and rarefied mean number of alleles did not differ significantly between not‐threatened and threatened groups of fishes in either freshwater or marine environments (Figure 3). Nevertheless, there was a trend in that the point estimates for the coefficients were smaller for all threatened versus non‐threatened fishes, suggesting that increased sample sizes or greater numbers of species may be needed to detect a small, but significant, effect. Although previous studies have found reduced genetic diversity in species of conservation concern relative to not‐threatened species (Spielman, Brook, & Frankham, 2004; Willoughby et al., 2015), our incorporation of a taxonomic correction of genetic data (via the PGLS model) more accurately accounted for the similarity in genetic diversity values due to taxonomic relatedness compared to analyses that did not use this approach (e.g., Willoughby et al., 2015), again suggesting that any differences, if real, would be driven by a small effect size. In addition, a reduction in population size (i.e., a population bottleneck) has to be sufficiently severe to result in decreased genetic diversity (Luikart, Sherwin, Steele, & Allendorf, 1998; Nei, Maruyama, & Chakraborty, 1975). Species can be listed as threatened (i.e., vulnerable, endangered, or critically endangered) by the IUCN if populations decline by ≥50%–90% over ten years or three generations, and decreases of this magnitude may be insufficient to constitute a true population bottleneck (IUCN, 2018). Alternatively, species can be listed as threatened in the event of a severe decline in habitat range size or quality, which may not result in a population bottleneck. As a result, fishes listed as threatened using IUCN criteria will not necessarily exhibit lower levels of genetic diversity than non‐threatened species.

**Figure 4:**
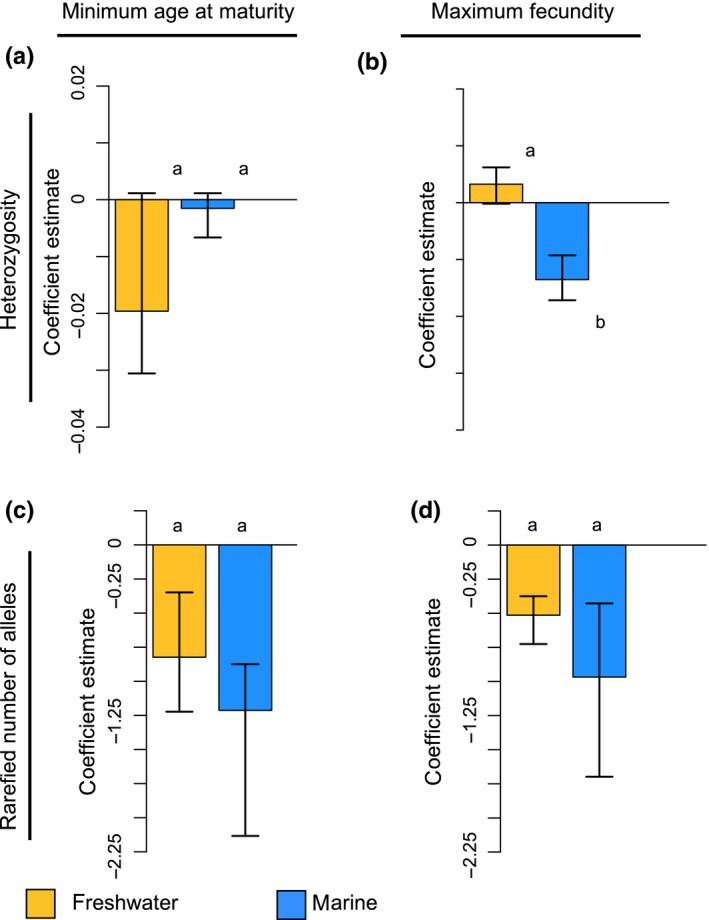
Bootstrapped phylogenetic least squares regression coefficients of mean heterozygosity and allelic diversity in freshwater and marine fishes in the class Osteichthyes estimated for two life‐history variables: age at maturity (a and c) and fecundity (b and d). In this figure, the coefficient estimates from the PGLS conservation status models represent the slope of the regression line where each life‐history trait was regressed against each measure of genetic diversity. Error bars represent 95% confidence intervals generated via bootstrapping. Significant relationships (nonoverlapping CIs between conservation status groups within each habitat) are represented by distinct letters (i.e., a and b). These models illustrate that minimum age at maturity and maximum fecundity are, for the most part, negatively related to genetic diversity in fishes (i.e., genetic diversity decreases as both minimum age at maturity and maximum fecundity increase)

We also found that minimum age at maturity was negatively related to genetic diversity in fishes (Figure 4). The observed negative relationship between age at maturity and genetic diversity supports our prediction that delayed age at maturity, a characteristic often associated with K‐strategist species, should decrease genetic diversity. Furthermore, our results are in agreement with other findings demonstrating a negative correlation between age at maturity and allozymic heterozygosity in populations of bony fishes (Mitton & Lewis, 1989; Nevo, 1978). More generally, our findings provide additional evidence of a negative relationship between age at maturity and genetic diversity across a broad range of taxa (Ellegren & Galtier, 2016; Romiguier et al., 2014). While the mechanisms underlying the observed negative relationship between age at maturity and genetic diversity require further investigation, one possible explanation is that late‐maturing species are characterized by a smaller effective number of breeders per generation, thereby intensifying genetic drift and resulting in reduced genetic diversity for species that reach maturity later.

Finally, we predicted that increasing fecundity should increase genetic diversity in fishes. However, contrary to our prediction, our results instead demonstrate a negative relationship between fecundity and genetic diversity in fishes (Figure 4). Thus, as fecundity increases, genetic diversity decreases. Our findings contradict both previous empirical findings regarding the relationship between fecundity and allozymic diversity in fishes and the positive relationship demonstrated between genetic diversity and fecundity for a broad range of invertebrate and vertebrate taxa (Mitton & Lewis, 1989; Romiguier et al., 2014). In our dataset, highly fecund fishes tend to be marine species that experience high variance in reproductive success through broadcast spawning, a phenomenon known as sweepstakes effects (Christie, Johnson, Stallings, & Hixon, 2010; Hedgecock, 1994; Pusack, Benkwitt, Cure, & Kindinger, 2016), which can greatly reduce *N_e_*, $\frac{N_{e}}{N}$, and therefore genetic diversity. Given that highly fecund marine species typically experience high variance in reproductive success (Hedgecock & Pudovkin, 2011), a positive relationship between fecundity and genetic diversity may be less likely. While broadcast spawning is utilized by some freshwater fishes, it is far more common in marine species (Hedgecock, 1994; Hoagstrom Christopher & Turner Thomas, 2015) perhaps at least partially explaining the more negative coefficients found in this group. Our findings illustrate that fishes represent a unique exception to the broadly observed positive relationship between fecundity and genetic diversity.

### 4.1 Future work

Future studies examining the factors that drive patterns of genetic diversity in fishes should investigate alternative markers and measures of genetic diversity (*e.g.,* nucleotide diversity), as increasing evidence suggests that population‐level processes affect different classes of genetic loci differently (Grant & Bowen, 1998; Palumbi & Baker, 1994; Zhang & Hewitt, 2003). Out of necessity, our study analyzed patterns of genetic diversity by collating measures of mean heterozygosity and rarefied number of alleles per locus from putatively neutral genetic markers. However, as the costs of next‐generation sequencing continue to decrease and sequencing data becomes more readily available, studies can begin to investigate whether patterns of genome‐wide genetic diversity in fishes exhibit similar relationships with factors investigated in our study (*e.g*., habitat, conservation status, and life‐history characteristics). Finally, while our study utilized a taxonomic correction to account for trait similarities due to shared evolutionary history, advances in our understanding of phylogenetic relatedness among fishes will improve as genomic data continues to emerge for new species. Based on the trends we uncovered, we expect that future studies can capitalize on well‐resolved phylogenies and thereby remove additional noise from the analysis; such studies may find stronger and perhaps different drivers of genetic diversity in fishes.

### 4.2 Applications to conservation

IUCN conservation rankings are the preeminent worldwide conservation ranking system used to categorize species based on risk of extinction. However, our results suggest that IUCN conservation status is a poor indicator of genetic diversity in fishes. Instead, we found that incorporating species‐specific habitat and life‐history information can improve our ability to discern fish species that exhibit reduced genetic diversity. As anthropogenic impacts continue to occur, rapid habitat alteration will present species with novel challenges. While we examined putatively neutral genetic markers, several studies have highlighted positive correlations between neutral markers and fitness or fitness‐related traits, suggesting that increased genome‐wide genetic diversity may also increase viability for populations facing environmental change (Chapman et al., 2009; Coltman & Slate, 2007; Forstmeier et al., 2012; Holderegger, Kamm, & Gugerli, 2006; Reed & Frankham, 2003). Thus, understanding factors that shape patterns of genetic diversity in fishes, including habitat and life‐history strategies, offers conservation managers an additional tool for predicting if and how species might adapt and respond to continued global change.

## CONFLICT OF INTERESTS

None declared.

## AUTHOR CONTRIBUTIONS

ASM, JRW, and MRC conceived the questions and objectives associated with the study. ASM and JRW compiled and analyzed the data. All authors contributed to the writing of the manuscript.

## DATA ACCESSIBILITY

Data utilized in this study are available in Supporting information Appendix S1.
