# Supplementary material for: Genetic diversity in fishes is influenced by habitat type and life‐history variation
Source: Ecol Evol. 2018 Nov 11;8(23):12022–31. doi: 10.1002/ece3.4661 (PMC6303716; doi:10.1002/ece3.4661)
Supplement: Supplementary file 2 [file ECE3-8-12022-s002.docx]

**Supplementary Figures**

**Fig. S1** Absolute value of the mean difference between the total number of alleles reported by each study and the mean number of alleles sampled across 100 sampling iterations of our rarefaction procedure as a function of the number of alleles tested for two closely related species in our data set. For some studies, the reported mean number of alleles was very close to the rarefied mean number of alleles (A), which typically happened for studies with large sample sizes, while in other studies the difference between the reported and rarefied mean number of alleles was larger (B). For this example, study-specific sample sizes were equal to 191 for *Haplochromis phytophagus* (A) and equal to 10 for *Haplochromis laparogramma* (B).

**Fig. S2** Relationship between total number of individuals sampled in a given study versus both the mean number of alleles per locus observed (A and C) and the mean rarefied number of alleles per locus (B and D) when sub-setting our data set for studies with maximum sample sizes less than or equal to 100 (A and B) or 1000 (*i.e.*, the entire data set; C and D). Each point represents a single study included in our data set. For each panel, a line of best fit generated from running a linear regression is plotted along with the associated model formula (R Core Team, 2017). These regression models demonstrate that our allele rarefaction procedure effectively increases the y-intercept and reduces the positive association between sample size and the number of alleles sampled in a given population Thus, the rarefication procedure effectively accounts for bias introduced by studies with small sample sizes and moderate to high levels of genetic diversity.

**Fig. S3** Bootstrapped phylogenetic least squares regression coefficients of mean heterozygosity and allelic diversity in freshwater and marine fishes in the class Chondrichthyes estimated for two life history variables: age at maturity (A and C) and fecundity (B and D). Error bars represent 95% confidence intervals generated via bootstrapping. Significant relationships are represented by CIs that don’t overlap zero for a given estimate. Significant differences between fishes from freshwater and marine environments in the relationship between genetic diversity and a given life history trait are represented by non-overlapping CIs and are represented by distinct letters (*i.e.*, a and b). These models suggest that minimum age at maturity and maximum fecundity are positively related to genetic diversity in this group of fishes, but it should be noted that small sample sizes (n = 32 species subdivided across three habitat types) resulted in overdispersion.

**Fig. S4** Mean genetic diversity estimates across families of fishes from different habitats. Mean heterozygosity (A) and mean number of alleles per locus (not rarefied) (B) are represented across families of fishes for all families with at least 3 species in our data set. Phylogenetic relatedness is indicated by the tree (C), where the number of species in each family is noted at each branch tip in parentheses after the family name. Median genetic diversity across species within each habitat type are represented by dashed lines. Notice that y-axis scale is smaller than the scale used in Figure 1.
